# The role of the physician associate in the United Kingdom

**DOI:** 10.1016/j.fhj.2024.100132

**Published:** 2024-04-20

**Authors:** Tomas Ferreira

**Affiliations:** Bristol Medical School, University of Bristol, Bristol, United Kingdom

**Keywords:** Physician Associates, NHS, Healthcare workforce, Patient safety

## Abstract

Physician associates (PAs) were introduced in the United Kingdom to address staffing shortages and fill service gaps, aiming to unburden doctors. Notably, in the backdrop of recent high-profile cases involving professional negligence and misconduct by PAs, the government has outlined a plan to expand the PA workforce and broaden their scope of practice. This commentary critically assesses the role, training, and regulation of PAs, juxtaposing the UK's approach with the US model. Concerns regarding disparities in training between PAs and doctors, potential impact on patient care quality, and lessons from the US experience raise substantial questions. Recommendations are provided to align with patient safety, professional standards, and the unique demands of the National Health Service (NHS). Despite the government's efforts to expand the PA workforce and its scope, uncertainties persist regarding their contribution to patient care and the implications for medical profesionals.

## Background

Physician associates (PAs), formerly assistants, were introduced in the United Kingdom (UK) in 2003 as a response to staffing shortages and the need to bridge service gaps, aiming to alleviate the burden on doctors.^1^ However, the role of PAs has come under increased scrutiny following high-profile professional negligence and misconduct cases.^2^^,^^3^ Incidents such as illegal prescription practices by PAs, including the unauthorised ordering of controlled medications and ionising radiation procedures, have further fuelled concerns about the government's plans to expand their numbers and scope within the NHS.^4^, ^5^, ^6^, ^7^ In light of these developments, a recent survey by the British Medical Association (BMA) has revealed significant concerns among doctors regarding the integration of PAs, as 87% of doctors perceived a risk to patient safety and 55% reported that PAs made their job more challenging rather than easier.^8^^,^^9^ This article seeks to critically evaluate the role of PAs within the UK healthcare system by analysing their education, training, economic considerations, integration challenges, and insights from the United States (US) model. Additionally, it provides recommendations to align their integration with healthcare system objectives.

## Training and qualifications

To train as a PA in the UK, candidates must hold an undergraduate degree, with a minimum classification of 2:2.^10^ The PA qualification program spans 2 years, including a mandatory minimum of 1,600 clinical hours, 200 of which can be in simulation.^11^, ^12^ Candidates also take the Physician Associate National Examination (PANE), a 200-question multiple-choice online exam, and a 14-station objective structured clinical examination (OSCE).^1^ In contrast, doctors must complete a minimum of 5,500 clinical hours, including a year of medical practice, to attain full professional registration.^13^ This significant disparity in clinical experience and training raises concerns about the depth of understanding and capability, particularly considering that some institutions report a 100% pass rate for PA exams.^14^

Moreover, beyond the quantitative differences in clinical training hours and examination rigour, there is a qualitative difference in the level and educational scope between doctors and PAs. Medical training is comprehensive, covering a wide range of medical conditions with both breadth and depth, and is designed to cultivate students into independent, autonomous practitioners with strong critical thinking and decision-making skills. In contrast, PA training focuses on foundational knowledge and clinical skills necessary to *assist* doctors in patient care. This discrepancy in the depth and breadth of their training raises critical questions about the roles and responsibilities that PAs can and should assume within the NHS. Unlike doctors, PAs are not subjected to any further postgraduate exams beyond their 2-year qualification. Additionally, there is currently no professional regulation governing PAs in the UK. This lack of stringent oversight, combined with differences in educational paths underscores the need for careful consideration of the role and regulation of PAs. While PAs indeed require significantly less training compared to doctors, this difference in training level corresponds to a discrepancy in qualifications. In healthcare, expertise is indispensable for ensuring patient safety. Thus, shorter training periods cannot be considered advantageous when patient safety and care quality are at stake.

Currently, a PA's scope of practice is often defined locally and depends significantly on the supervising physician, relying on self-reported competence assessments or a trial-and-error approach. This variability and lack of formal standardisation not only highlight a critical gap in ensuring consistent and safe integration of PAs into the healthcare workforce but also raise concerns about the potential for inconsistent patient care quality and increased risk in clinical settings.

## The US experience

The UK's adoption of the PA role was inspired by the US, which originally introduced PAs in the mid-20th century to improve healthcare accessibility in underserved areas and generate profit.^15^ Projections indicate that the number of PAs in the US will grow by 31.3% between 2019 and 2029, while the growth rate for physicians and surgeons stands at only 3.6%. Consequently, the physician-to-PA ratio is expected to decrease from 6:1 to 4.7:1 during this period. This shift is attributed to several factors, including legislative changes, profit-driven actions by for-profit health institutions, and a substantial pay gap between doctors and PAs.^15^ Notably, growing tension among US medical professionals, exemplified by organisations such as the American Medical Association (AMA) consistently opposing non-doctor-led care, underscores the concerns surrounding PA expansion.^16^

However, this profit-seeking approach has also revealed significant shortcomings. Research from the US highlights that doctors outperform PAs in minimising diagnostic and treatment malpractice.^17^ Moreover, studies find that the average PA overprescribes opioids at more than twice the rate of doctors.^18^ Further, patient preferences tend to favour physician-led care, highlighting the perceived importance of a physician's education and training.^19^ As the UK considers expanding the role of PAs, lessons from the US highlight the need for stringent oversight and regulation to avoid similar pitfalls. The economic incentives shaping the PA model in the US do not correspond to the objectives and principles of the NHS's not-for-profit healthcare system. Therefore, the implementation of the PA model within the NHS must be carefully considered and tailored to align with the unique demands and ethical commitments of healthcare provision in the UK. Despite the government's desire to cut costs, it must not compromise quality of care and patient safety. The US experience with PAs should serve as a cautionary tale rather than a template, alerting policymakers and practitioners to potential pitfalls and limitations.

It is also worth noting that the US physician assistant role significantly differs from that in the UK. In the US, PAs are required to accumulate extensive clinical experience hours *before* even applying to a PA course, and have a well stipulated scope of practice.^20^ However, recently, even the US PA role has faced scrutiny, with data indicating that patients should never be solely managed by non-doctors.^21^

## Developments within the UK healthcare system

Recently, Royal Colleges, namely Ophthalmology and Psychiatry, have announced pilot schemes of PAs in their specialty, funded by Health Education England (HEE).^22^^,^^23^ These initiatives were seemingly implemented without clear member consultations, who are not only the financial backers of the colleges but also the professionals who will be expected to supervise these PAs. While the intention behind the integration of PAs may be to alleviate the workload of doctors, the added responsibility of supervising less-trained staff could paradoxically increase the doctors' workload, as alluded to by the recent BMA survey.^9^ This raises questions about the effectiveness of PAs in actually unburdening doctors. The situation is further exacerbated by the significant – and increasing - training bottlenecks within medical specialties.^24^ With a thousands of doctors, unable to obtain a training post, and vying for limited positions, introducing funds for non-doctor roles seems misaligned with the current challenges the system faces. This is particularly pronounced in the field of ophthalmology, where, despite a pressing demand, HEE funded a mere 78 specialty training places nationwide.^24^^,^^25^ Such decisions not only risk compromising the quality of specialist care but also overlook the vast pool of highly trained doctors waiting to contribute to their chosen fields.

Further, we must also consider the implications for Locally Employed (LE) and Specialty and Associate Specialist (SAS) doctors. These medical professionals, integral to the NHS, are faced with challenges in supervision, training, and career progression similar to those faced by PAs. However, the narrative surrounding PAs frequently underscores these challenges to rationalise their remuneration and roles, overshadowing the parallel realities of LE/SAS doctors. It is important to ensure that integration of PAs does not come at the expense of LE/SAS doctors' opportunities and professional development.

## Scope creep, implications, and the burden of proof

In the healthcare context, `scope creep' refers to the gradual expansion of roles and responsibilities beyond originally defined boundaries. For non-medical professionals such as PAs, this may lead to undertaking tasks traditionally performed by doctors. The potential dangers of this include confusion over responsibilities, potential dilution of patient care quality, and potential legal ambiguities regarding accountability. These concerns have been previously noted.^26^

Unlike general practitioners (GPs), who are trained to provide holistic assessments, there is an indication that PAs may tend towards more compartmentalised evaluations. For instance, PAs may approach patients by addressing symptoms via protocol-driven, isolated diagnostic and treatment pathways, while overlooking underlying conditions. Instances where patients present with common symptoms—such as fatigue, sweating, or tearfulness—might result in targeted interventions that fail to identify underlying issues, such as menopause. This underscores the inherent challenge in discerning the `simplicity' of a patient's case and whether it is appropriate for patients to see a PA over a GP; what appears superficially straightforward can rapidly evolve into a complex clinical scenario.

This integration of non-medical professionals such as PAs into tasks traditionally undertaken by doctors raises questions about whether condensed training can equate to the extensive experience and education of doctors. It also raises questions about the potential loss of care quality and expertise. Demonstrating the safety, efficacy, and quality of care provided by PAs requires robust evidence, and the responsibility to provide this evidence should rest with policymakers advocating for PA integration, rather than requiring detractors to emphasise potential risks. Furthermore, it is critical to identify the distinct value that the integration of PAs brings to the multidisciplinary team (MDT) that might not be fully provided by other existing roles. Understanding these unique contributions is essential in avoiding redundancy, role confusion, diluted care, or inefficiencies within the system.

In recent discussions about the role of PAs within the NHS, there has been an increasing rhetoric about the benefit of PAs in addressing 'simpler' patient cases. This approach aims to allow doctors to concentrate on more 'complex' cases. This strategy, while seemingly efficient, overlooks the implications for doctors who then face a relentless stream of `complex' patients. Such a scenario, often referred to as 'working to the top of your licence,' can be professionally overwhelming, increasing the risk of burnout among doctors.

Another critical aspect is the loss of training opportunities for doctors. As doctors are required to rotate departments, or even trusts, and PAs are not, PAs may be able to build stronger relationships with departments and department management. This can lead to them being prioritised in training opportunities and procedures that would often fall under the remit of doctors and not allied professionals. It highlights a contention against decisions that undervalue doctors, raising potential risks to patient care. Advocates for PAs frequently argue that they offer continuity of care. This argument could be nullified through the restructuring of the rotational system for doctors, suggesting that the unique benefit attributed to PAs in this context is neither robust nor exclusive.

Finally, the primary goal of all decisions and changes in the healthcare landscape should be to prioritise patient care quality. Any shift in roles, responsibilities, or practices must be rigorously assessed against this benchmark, ensuring that the focus remains on delivering optimal patient care.

## Recommendations

Based on the comprehensive evaluation of the role, challenges, and implications of PAs within the NHS, the following recommendations are offered to improve patient care, maintain professional standards, and ensure a balanced integration of PAs within existing medical frameworks.

- **Independent evaluation**: It is critical for independent research bodies to conduct impartial, peer-reviewed studies assessing the effectiveness and scope of PAs in the UK healthcare system, in order to inform evidence-based policy decisions. Current research advocating for the utility of PAs often suffers from significant conflicts of interest.^27^ The majority of these studies are conducted by PAs themselves, or by individuals who stand to gain financially from the expansion of the PA role, such as educators in PA programmes or employers in private practice. Such conflicts may skew the findings and conclusions, calling for an urgent need for impartial, independent evaluations to objectively assess the role and efficacy of PAs.
- **Consultative approach**: Royal colleges and professional bodies must urgently consult with their members, before introducing or expanding the roles of PAs within their specialties. This consultation will ensure decisions align with the insights and experience of healthcare professionals actively involved in patient care and the supervision of these individuals. Recently, the Royal College of Physicians (RCP) and the Royal College of Anaesthetists (RCoA) have held Extraordinary General Meetings (EGMs) discussing how to tackle the expanding role of PAs (or anaesthesia associates (AA)).^28^^,^^29^ Both colleges overwhelmingly called for an immediate pause for all PA and AA role recruitment and expansion. The Royal College of Physicians of Edinburgh has also recently announced that they believe there should be no further expansion of the PA workforce in the UK, and there should be a pause in recruitment of PAs within the NHS.^30^ This indicates a rising concern within the medical community and underscores the need for a consultative approach involving all stakeholders.
- **Economic analysis**: Acknowledge ongoing health economic evaluations to determine whether PAs provide value for money within the NHS framework. It is essential to await the outcomes of these comprehensive studies before any further expansion is considered. These analysis should consider PAs’ higher salaries, when compared to medical graduates, and assess potential concerns about the quality of non-doctor healthcare delivery, which may increase the overall admission costs. Moreover, these analyses should extend to encompass potential indirect costs associated with PA-led care. This includes examining the costs of increased lengths of stay, higher readmission rates, and the potential for increased litigation costs. A recent economic analysis of AAs revealed the current operational model to be non-viable and economically detrimental to the NHS, a finding likely applicable to PAs as well.^31^ This underscores the importance of awaiting the outcomes of comprehensive health economic evaluations before further expansion is considered.
- **Scope regulation**: Implement a nationwide standardised scope of practice for PAs to prevent inconsistent practices across regions and hospitals. Clearly defined boundaries will aid in maintaining optimal patient care and safety for both PAs and patients, as well as mitigating tensions between healthcare professionals.
- **Protection for doctors in training**: It is critical to protect the training and career development opportunities for doctors. Measures must be implemented to safeguard and prioritise doctors' training and prevent compromise in this essential aspect of medical education. Given today's competitive specialty entry ratios,^21^ the rationale behind hiring PAs to address shortages that doctors are actively seeking to alleviate remains unclear.
- **Salary adjustments**: It is necessary to address the discrepancy in salaries between newly qualified PAs and doctors. PAs start with an annual salary of £43,742 after completing a 2-year qualification for a 37.5 h week, while a doctor in their first year after qualification earns £32,398 for a contracted 48 hours.^32^^,^^33^ This is despite the fact that doctors assume greater responsibilities, work night shifts, have on-call commitments, incur higher costs for education and professional fees, and actively supervise PAs. While some argue that doctors have a higher potential for salary growth, it is essential to consider that compensation should be commensurate with the responsibilities and qualifications required for the current role, rather than future earnings potential. Failure to address this discrepancy risks exacerbating inter-professional tensions and could negatively impact collaborative healthcare delivery.
- **Prescription and radiation authority**: Critically evaluate the prudence of permitting PAs to independently prescribe medications and order ionising radiation procedures. A strong emphasis should be placed on the requirement for evidence-based studies, rigorously assessing the safety and appropriateness of granting these significant medical responsibilities to PAs, particularly in light of their training.
- **Re-evaluation of the 'Physician Associate' title**: The name 'physician associate' has inherent ambiguities which can lead to misconceptions about the PA's role and capabilities among patients. This confusion is further compounded when PAs introduce themselves using ambiguous terminology without explicitly stating their qualifications. This lack of transparency can not only mislead patients into believing they have been consulted by a doctor but may also compromise the integrity of informed consent. By obscuring the true nature of the care being provided, patients may be deprived of the opportunity to make fully informed decisions about their treatment. It is essential to re-examine this title and consider alternatives that provide clarity about the PA's qualifications and scope of practice. The current title does not make it clear that the title holder can only practise under *direct* supervision from a doctor. A shift towards using the term *'assistant'* could be a valuable starting point; however, the use of *'physician'* in the title also merits re-examination.
- **Clarification of litigation and responsibility**: The accountability dynamics between PAs and the doctors supervising them remain ambiguous. There is a pressing need to establish clarity around who bears responsibility when errors occur. This becomes particularly significant given that doctors often find themselves supervising PAs without being explicitly consulted about it. Clear guidelines should be established detailing the liability lines. If doctors are expected to shoulder the responsibility for decisions or errors made by PAs under their supervision, then they must be involved in the decisions around the integration and supervision of PAs in clinical settings. To this end, the BMA has helpfully provided guidance regarding the scope of tasks in which doctors should agree to supervise PAs.^34^
- **Regulation**: The regulation of PAs is essential for ensuring oversight, professional responsibility, and accountability within the healthcare system. However, the current plan for the GMC to regulate PAs is unsuitable, as the GMC's mandate is specifically to oversee doctors. Assigning the GMC this additional role would further blur professional boundaries. A more appropriate body for such regulatory oversight would be the Health & Care Professions Council, which already has experience regulating other medical associate professions. Importantly, the focus of any regulatory framework should be on maintaining standards and accountability, rather than serving as a conduit for expanding the scope of PAs.
- **Transparency and stakeholder perspectives**: Policy decisions regarding PAs must consider the views of various stakeholders, including patients, and other healthcare professionals. Public consultations, surveys and targeted interviews can provide insights into preferences and concerns that may shape public opinion. This approach ensures that decisions regarding PAs align with societal values, patient satisfaction levels, and the overall efficiency and quality of care within the NHS. It fosters transparency and trust, guiding policy in a way that respects those most directly impacted. Moreover, there is a need for public education to clarify the roles of various professionals in the healthcare system, including PAs. This is important to ensure that patients are aware of the credentials of those providing their care and can make informed choices.

In addressing the complexities surrounding the integration and use of PA within the NHS, it is important to acknowledge the individual journeys and commitments of PAs who may have entered the profession with genuine intentions to contribute positively to patient care. Many PAs come from diverse backgrounds, with some having faced socioeconomic barriers that may have precluded the pursuit of traditional medical education. Others may have been motivated by the prospect of a potential 'transition' into medicine. It is clear that the challenges we face are not just systemic but deeply personal to those within the PA profession. The discourse around the role of PAs should, therefore, extend compassion and recognition to these individuals who have been equally let down by the inadequacies of the system. However, it is also evident that PAs may have emerged as an inadequate and potentially unsafe solution to issues stemming from chronic underfunding of the NHS.

## Conclusion

In conclusion, the introduction of PAs in the UK, alongside the recent NHS Long Term Workforce Plan's proposal to increase their numbers and expand their scope requires careful consideration. While these changes aim to address staffing shortages, they raise concerns regarding training disparities, potential impacts on patient care quality, and economic implications. Drawing valuable insights from the US experience, it becomes evident that a cautious and tailored approach is essential. It is unclear what unique role PAs can fulfil within an MDT, as it is challenging to discern their distinct contributions compared to other MDT members. Given the current competition ratios for entry into specialty training, questions arise regarding the rationale behind hiring PAs to address shortages that doctors are actively seeking to alleviate. The recommendations presented here aim to align this integration with professional standards, patient safety, and efficient healthcare delivery. The discourse surrounding PAs must remain rigorous and patient-centric, acknowledging the complexities and challenges of their role within the UK healthcare system.
